# P-126. Complete device extraction optimizes cure in deep brain stimulator and responsive neurostimulator infections: a single center cohort

**DOI:** 10.1093/ofid/ofaf695.353

**Published:** 2026-01-11

**Authors:** Tyler Rosengren, Nicolas W Cortes-Penfield, Josue Avecillas-Chasin, Aviva Abosch

**Affiliations:** University of Nebraska Medical Center, Omaha, Nebraska; University of Nebraska Medical Center, Omaha, Nebraska; University of Nebraska Medical Center, Omaha, Nebraska; University of Nebraska Medical Center, Omaha, Nebraska

## Abstract

**Background:**

Infection is a serious yet ill-studied complication of implanted neurostimulator therapy. We reviewed the epidemiology and management of neurostimulator infections at a large Midwestern academic medical center.

**Figure:**
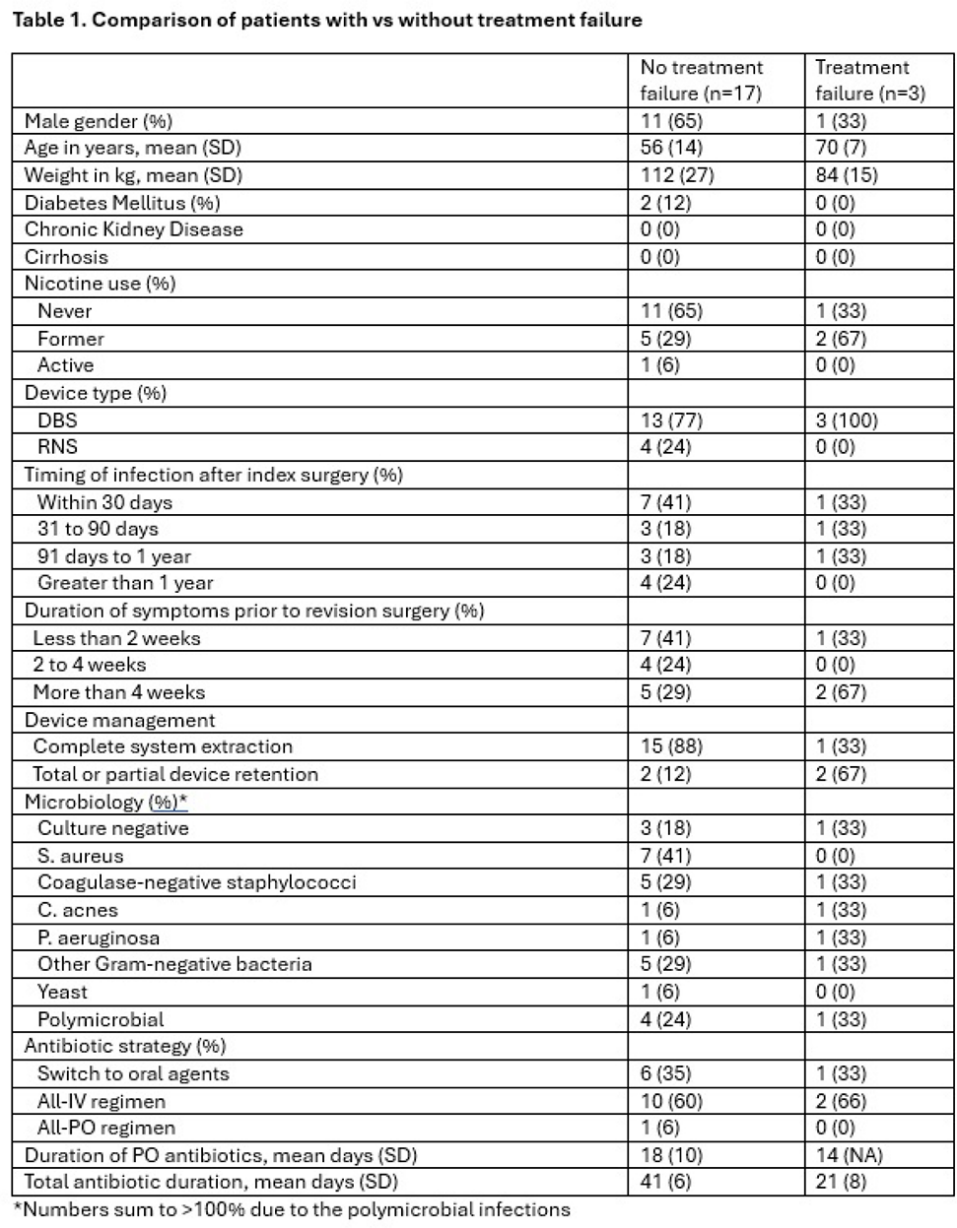

**Methods:**

We reviewed all adults admitted to the University of Nebraska Medical Center from January 1^st^ 2012 to August 1^st^ 2024 who underwent surgery related to a deep brain stimulator or responsive neurostimulation device (DBS or RNS; surgeries identified by procedure code) for proven or suspected infection (identified by review of the neurosurgery and infectious disease teams’ clinical documentation) followed by at least two weeks of antibiotics. We defined treatment failure as having had death from any cause, unplanned reoperation for infection, or antibiotic retreatment, assessed at 90 days and one year from the initial surgery for infection.

**Results:**

We identified twenty patients; 16 had DBS infections, their median age was 61 years, and their median weight was 103kg. Device indications included Parkinson’s (50%), essential tremor (20%), seizures (20%), and dystonia (10%). Infections mostly occurred within one year of device placement and the majority had indolent onset. *S. aureus* (35%), Coagulase-negative staphylococci (30%), and non-*Pseudomonas* Gram-negatives (30%) were the most common pathogens; 25% of infections were polymicrobial.

Three patients (15%) experienced treatment failure at 90 days, including one death, one unplanned reoperation for infection, and one antibiotic retreatment without surgery. There were no additional failures at one year. Complete device explantation was performed in 80% of cases and was associated with fewer treatment failures (6% vs 50% with debridement alone or incomplete device removal). Switch to oral antibiotics was used in 35% of cases and not associated with treatment failure. The median antibiotic duration was 42 days; 2/3 failures occurred during antibiotic therapy.

**Conclusion:**

In neurostimulator infections, complete device extraction is associated with fewer treatment failures than less aggressive surgeries. Our findings are limited by small sample size; larger multi-institutional studies are needed.

**Disclosures:**

Aviva Abosch, MD PhD, Medtronic: Advisor/Consultant|Medtronic: Grant/Research Support

